# Identification, Screening and Mechanism Analysis of Anti-Parkinson’s Disease Peptides from *Rapana venosa* Protein Hydrolysates

**DOI:** 10.3390/md24050180

**Published:** 2026-05-15

**Authors:** Qingzhong Wang, Shuqin Shao, Yizhuo Wang, Wenshuai Fan, Zilong Wang, Xuchang Liu, Kechun Liu, Shanshan Zhang

**Affiliations:** Biology Institute, Qilu University of Technology (Shandong Academy of Sciences), Jinan 250103, China; 17852607519@163.com (Q.W.); 15206871973@163.com (S.S.); 15552566116@163.com (Y.W.); 15737566970@163.com (W.F.); 13475429667@163.com (Z.W.); liuxuchang@sdfmu.edu.cn (X.L.); liukc@sdas.org (K.L.)

**Keywords:** *Rapana venosa*, peptide, anti-Parkinson’s disease activity, zebrafish, molecular docking, Transcriptome sequencing

## Abstract

At present, there is still a lack of effective treatments to slow the progression of Parkinson’s disease. Naturally derived active substances, valued for their safety and multi-target potential, have become an important direction in anti-PD drug development, with marine organisms representing a valuable source of bioactive peptides. This study aimed to isolate and identify anti-PD peptides from *Rapana venosa* protein hydrolysates. Through bioactivity-guided screening combined with an MPTP-induced zebrafish PD model, three novel active peptides—KSTELLI, FLVKLPMFM, and SDSLSEILIS—were successfully identified. The study showed that these peptides significantly alleviated dopaminergic neuron loss, improved the cerebral vascular system, restored motor and sensory function, and alleviated oxidative stress. Molecular docking confirmed their stable binding to key PD targets (DDC, α-synuclein, and MAO-B). Further transcriptomic and gene expression analyses revealed that their neuroprotective effects involve the regulation of pathways related to metabolism, oxidative stress, inflammation, and apoptosis, with the three peptides exhibiting distinct mechanistic emphases. The research demonstrates that these marine-derived peptides exert neuroprotective effects through a synergistic multi-target mechanism, laying a foundation for the development of novel lead compounds against Parkinson’s disease.

## 1. Introduction

Parkinson’s disease (PD) is a progressive neurodegenerative disorder primarily affecting the elderly [1], characterized by the loss of dopaminergic neurons in the substantia nigra and the aggregation of α-synuclein in Lewy bodies [2]. These pathological changes lead to dopamine deficiency in the striatum, resulting in both motor symptoms (e.g., resting tremor, bradykinesia, rigidity) and non-motor symptoms (e.g., cognitive impairment, autonomic dysfunction), which progressively worsen over time [3,4,5]. Currently, there is no cure for PD [6]. Clinical treatments, such as levodopa and dopamine agonists, only alleviate symptoms and are often accompanied by significant side effects with long-term use, without halting disease progression [7]. Therefore, the development of safe, effective, and multi-target neuroprotective agents remains an urgent need.

Marine organisms represent a vast and underexplored reservoir of such bioactive peptides. Bioactive peptides derived from food sources have gained attention for their neuroprotective potential, owing to their safety, biocompatibility, ability to cross the blood–brain barrier, and multi-target mechanisms [8]. These peptides can mitigate key pathological processes in PD, including oxidative stress, neuroinflammation, mitochondrial dysfunction, and metabolic dysregulation [9,10,11]. In particular, peptides that simultaneously enhance antioxidant capacity and regulate cellular metabolism are promising candidates for intervening in the complex pathology of PD.

The marine snail *Rapana venosa* is abundant in various nutrients and bioactive compounds, providing it with high edible and medicinal value [12,13]. Studies have found that dietary intake of *Rapana venosa* can significantly enhance the lipid metabolism and antioxidant capacity [14]. The proteins and their enzymatic hydrolysate peptides derived from *R. venosa* exhibit notable bioactivities, including antioxidant [15], anti-inflammatory [13,16], immunomodulatory [17], and anti-tumor effects [18]. Furthermore, our research group previously identified bioactive peptides from *R. venosa* protein hydrolysates that exert anti-colitis activity by alleviating oxidative stress, mitigating inflammation, and inhibiting apoptosis [19,20]. The multi-faceted bioactivities of *R. venosa* peptides, which align with the core pathological pathways of PD (e.g., oxidative stress, neuroinflammation, and apoptosis), strongly suggest their potential as promising candidates for PD intervention. However, their efficacy and mechanisms in PD models remain largely unknown, and whether they can exert anti-PD effects through integrated modulation of oxidative stress and metabolism warrants systematic investigation.

The zebrafish (*Danio rerio*) has emerged as a powerful vertebrate model for PD research due to its genetic and neuroanatomical conservation with humans, particularly in dopaminergic systems [21,22]. The 1-Methyl-4-phenyl-1,2,3,6-tetrahydropyridine (MPTP)-induced zebrafish PD model recapitulates key features of the disease, including dopaminergic neuron loss, motor deficits, and molecular dysregulation in pathways related to oxidative stress, inflammation, and metabolism [23,24,25]. Combined with transcriptomic analysis, this model enables comprehensive evaluation of neuroprotective agents and elucidation of their mechanisms [26].

In this study, we employed an MPTP-induced zebrafish PD model to isolate and screen the potential neuroprotective peptides from *Rapana venosa* protein hydrolysates. Then the anti-PD effects of the identified active peptides were evaluated through a series of in vivo bioactivity assays. The underlying neuroprotective mechanisms of the three bioactive peptides derived from *R. venosa* protein hydrolysates were elucidated using an integrated approach combining transcriptomic and bioinformatic analyses with molecular biology validation. These findings provide a scientific foundation for the development of multi-target, marine-derived therapeutics for Parkinson’s disease.

## 2. Results

### 2.1. Isolation and Purification of Anti-Parkinson’s Disease Peptides from Rapana venosa Protein Hydrolysates

In this study, simulated gastrointestinal enzymatic digestion was used for the preparation of proteins from *Rapana venosa*. The enzymatic digestion efficiency was assessed by determining the degree of hydrolysis (DH) of protein from whole *Rapana venosa* tissue. Then, the hydrolysate was obtained with DH of 45.81%. The result indicates that large amounts of peptides were released from *R. venosa* protein.

**Figure 1:**
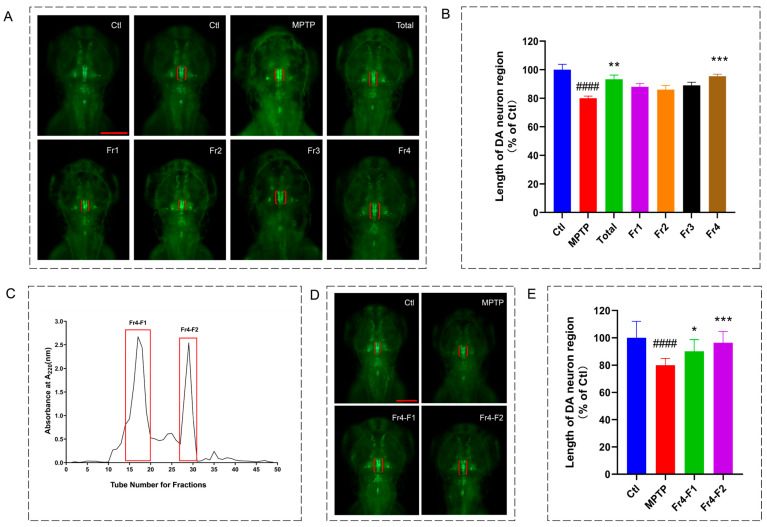
Neuroprotective effects of *Rapana venosa* protein hydrolysates and the purified peptide fractions on dopaminergic neurons in zebrafish. (**A**) Representative fluorescence images of the midbrain region in *vmat2:GFP* zebrafish from the control, MPTP model, and different ultrafiltration fraction-treated groups. Dopaminergic neurons are indicated by red brackets. Scale bar: 100 µm; (**B**) statistical analysis of the dopaminergic neuron region length. Data are expressed as a percentage of the control (*n* = 10). #### *p* < 0.0001 vs. control; ** *p* < 0.01 and *** *p* < 0.001 vs. MPTP group; (**C**) elution profile (absorbance at 220 nm) of the active ultrafiltration fraction (<3 kDa) further separated by a Sephadex G-25 gel filtration column; (**D**) representative fluorescence images of the midbrain region in *vmat2:GFP* zebrafish from the control, MPTP model, and gel-filtration purified fraction-treated groups. The red line is the scale bar: 100 µm; (**E**) Statistical analysis of the length of the DA neuron region in each group. Data are expressed as percentage of the control group (*n* = 10). #### *p* < 0.0001 vs. control; * *p* < 0.05 and *** *p* < 0.001 vs. the MPTP.

To further screen for anti-PD peptides from the hydrolysate, ultrafiltration membranes was used to separate the hydrolysate. Based on differences in molecular weight cut-off, the peptides were fractionated into four fractions: MW > 10 kDa (Fr1), 5–10 kDa (Fr2), 3–5 kDa (Fr3), and <3 kDa (Fr4). Subsequently, the anti-PD activity of the protein hydrolysates (Total) and each fraction was evaluated using an MPTP-induced Parkinson’s-like zebrafish model. By observing and comparing the effect of peptide on the development of dopaminergic neurons in zebrafish larvae, the experimental results demonstrated that, at the same concentration, the protein hydrolysates (Total) and the Fr4 fraction exhibited the most potent dopaminergic neuroprotective activity, capable of effectively improving neuronal morphology and promoting their growth (Figure 1A,B).

Thus, in order to obtain a more efficient fraction, the Fr4 part was purified using the Sephadex-G25 with the separation range of 1000–5000 Da. As shown in Figure 1C, Fr4 part was separated and purified into 2 fractions Fr4-F1 and Fr4-F2 by Sephadex-G25 column. In the present study, the Fr4-F2 fraction exhibited higher dopaminergic neuroprotective activity than F1 fraction (Figure 1D,E). Thus, the Fr4-F2 fraction was selected for further analysis.

### 2.2. Identification and Screening of the Potential Anti-Parkinson’s Disease Peptides

The certain peptides in Fr4-F2, which might show potent anti-PD activity, were further identified by UPLC-MS/MS. A total of 14 peptides were identified from Fr4-F2 fraction by comparing the data available in the NCBI database, and the 14 peptides ranged in length from 7 to 10 amino acids (Table 1).

To evaluate the neuroprotective potential of the identified peptides, molecular docking was performed against three key protein targets implicated in Parkinson’s disease: human dopa decarboxylase (DDC, PDB: 1JS3), human alpha-synuclein (α-syn, PDB: 3Q25), and human monoamine oxidase B (MAO-B, PDB: 4A79). As shown in Table 1, most of the peptides exhibited strong binding affinities with dopa decarboxylase and alpha-synuclein. Only TNLLMILLI (P14) exhibited a lower docking score with dopa decarboxylase, and only MILLGLVLMG with alpha-synuclein, compared to the positive control drug levodopa. In contrast, for the monoamine oxidase B target, only the peptides FLVKLPMFM (P3), LLIRAGL (P9), FGINLIQ (P2), NLLGLVL (P13), and GLEINLIGF (P4) showed higher docking scores than that of the positive control.

Subsequently, the potential bioactivity of the identified peptides was further evaluated using the BIOPEP-UWM database (https://biochemia.uwm.edu.pl/biopep/start_biopep.php, accessed on 12 January 2025). The search was specifically targeted towards known functional amino acid motifs implicated in anti-Parkinson’s mechanisms—including antioxidant, dipeptidyl peptidase IV inhibitory, neuropeptide, and metabolic regulatory sequences (detailed results are provided in Table S1). Among the screened peptides, FLVKLPMFM (P3), KSTELLI (P6), and SDSLSEILIS (P7) were found to harbor a higher prevalence of these bioactive fragments, indicating their stronger potential for anti-Parkinson’s activity.

**Figure 2:**
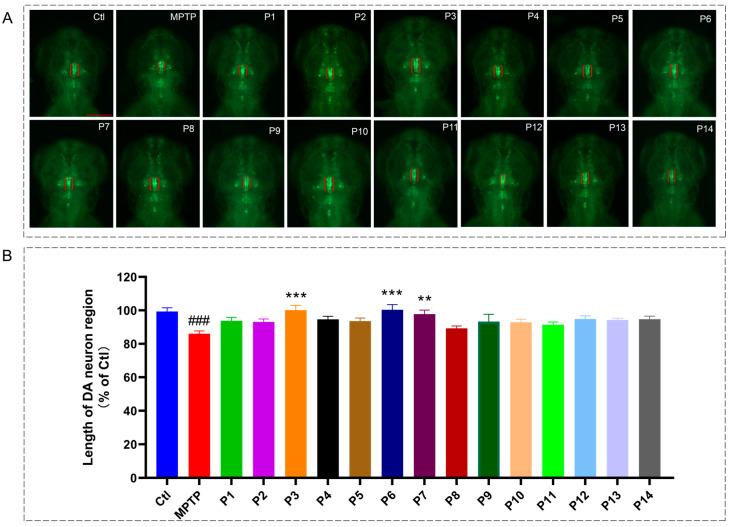
The protective effects of identified peptides on dopamine neurons in zebrafish. (**A**) Representative fluorescence images of the midbrain region in *vmat2:GFP* zebrafish from the control, MPTP model, and peptide-treated groups; Dopaminergic neurons are indicated by red brackets. The red line is the scale bar: 100 μm; (**B**) Statistical analysis of the dopaminergic neuron region length. Data are expressed as percentage of the control group (*n* = 10). ### *p* < 0.001 vs. control; l; ** *p* < 0.01 and *** *p* < 0.001 vs. the MPTP.

Furthermore, utilizing a zebrafish model of Parkinson’s disease, we evaluated the neuroprotective effects of the identified peptides. All 14 peptides were synthesized and subsequently assessed for their ability to protect dopaminergic neurons. As shown in Figure 2, FLVKLPMFM (P3), KSTELLI (P6), and SDSLSEILIS (P7) significantly alleviated MPTP-induced damage to midbrain dopaminergic neurons in zebrafish compared to MPTP group (*p* < 0.01). Thus, three synthesized peptides (P3, P6, and P7) were selected for further interaction analysis and bioactivity evaluation.

### 2.3. The Interaction Profiles of Selected Anti-PD Peptides and PD-Related Key Targets

**Figure 3:**
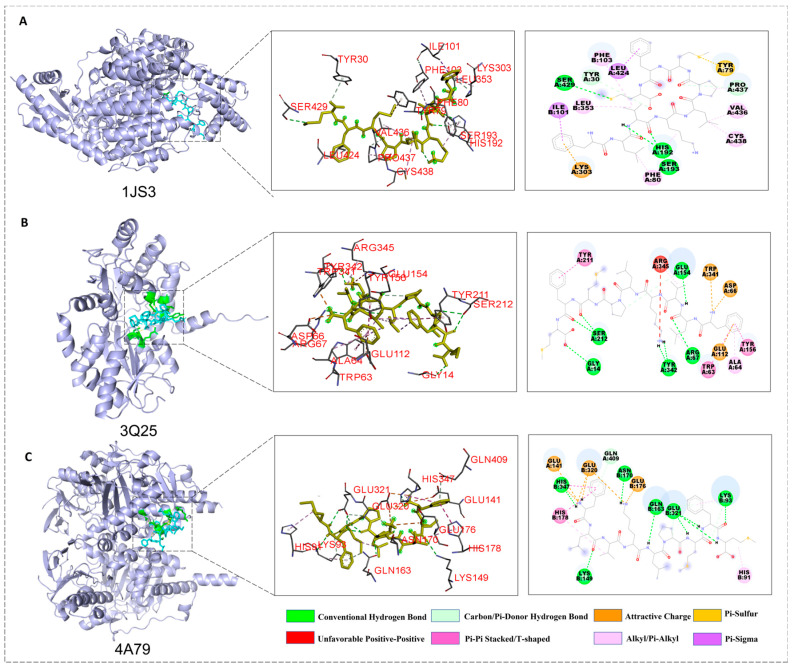
The molecular docking results for the peptide-receptor complex with the highest binding affinity. Each result contains an overall picture, a local zoomed-in picture and a 2-D diagram. (**A**) 1JS3 (DDC); (**B**) 3Q25 (α-syn); (**C**) 4A79 (MAO-B).

To elucidate the differences in the binding mechanisms between different ligands and target proteins, this study systematically compared the binding modes of the peptides FLVKLPMFM (P3), KSTELLI (P6), SDSLSEILIS (P7), and the positive control drug levodopa with the targets 1JS3, 3Q25, and 4A79 using two-dimensional interaction diagrams. This analysis revealed the structural basis for the high binding affinity of P3. Consequently, the detailed interaction diagrams of P3 with the key targets are displayed in Figure 3; the docking results for P6, P7, and levodopa are detailed in Figure S1.

Docking analysis with 1JS3 (DDC) indicated that FLVKLPMFM (P3) forms a synergistic hydrogen bond network through serine residues, with its conformation precisely anchored by secondary hydrogen bonds involving tyrosine and proline, and without producing unfavorable interactions. Its hydrophobic side chains established extensive alkyl/π-alkyl interactions with multiple residues and constructed π-sulfur and π-cation networks, resulting in the highest binding specificity. Although KSTELLI (P6) and SDSLSEILIS (P7) could also form rich hydrogen bonds, their overall stability was compromised by donor-donor repulsion or negative charge-negative charge repulsion, respectively; furthermore, their hydrophobic interaction networks were relatively simple. Levodopa had a limited hydrogen bond network, exhibited unfavorable repulsion, and possessed the weakest hydrophobic interactions and binding specificity.

Docking analysis with 3Q25 (α-syn) showed that all three peptides could form extensive hydrogen bond networks, with SDSLSEILIS (P7) having the most comprehensive network. Regarding hydrophobic/π interactions, FLVKLPMFM (P3) displayed the greatest diversity, including π-π stacking, T-shaped, and π-cation interactions, among others. KSTELLI (P6) and SDSLSEILIS (P7) primarily relied on alkyl/π-alkyl interactions. Levodopa formed only a single π-π stacking interaction, resulting in a significantly weaker interaction network compared to the peptides.

In the docking analysis with 4A79 (MAO-B), FLVKLPMFM (P3) once again formed a comprehensive hydrogen bond network covering both the backbone and side chains and exhibited the most diverse π-related interactions (such as π-cation and π-π T-shaped interactions). KSTELLI (P6) and SDSLSEILIS (P7) possessed stable hydrogen bond networks, but their hydrophobic interactions were mainly alkyl-based. Levodopa had the most limited hydrogen bonding and hydrophobic interactions.

The results indicate that the three peptides exhibit certain differences in their binding affinity for PD-related targets. Therefore, combining these findings with further animal experimental validation is necessary to explore the mechanisms of action of these three peptides.

### 2.4. Effect of Peptides on Dopaminergic Neuron Development in PD-like Zebrafish

**Figure 4:**
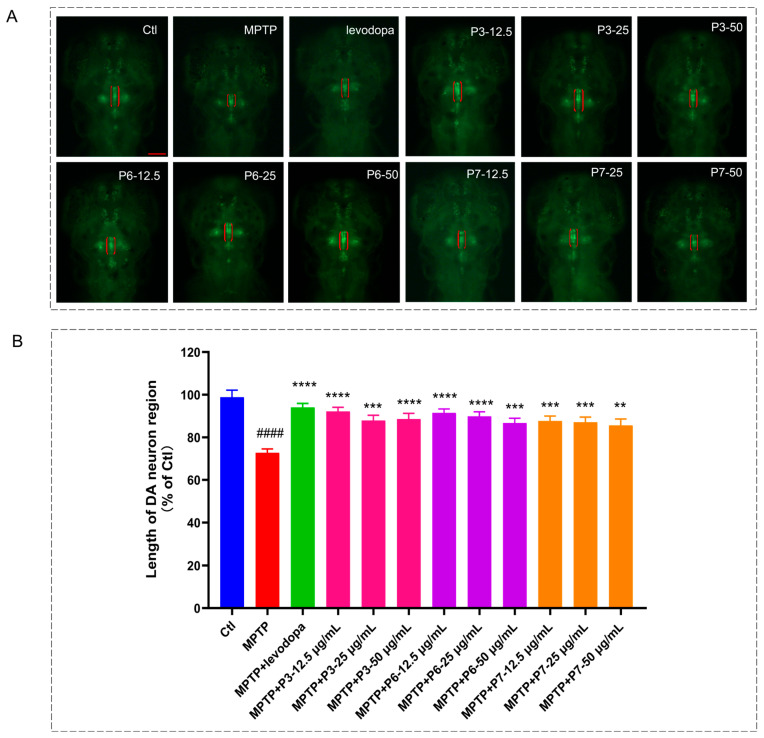
The protective effects of active peptides on dopamine neurons in zebrafish. (**A**) Representative fluorescence images of the midbrain region in *vmat2:GFP* zebrafish from the control, MPTP model, and peptide-treated groups; Dopaminergic neurons are indicated by red brackets. The red line is the scale bar: 100 µm; (**B**) statistical analysis of the dopaminergic neuron region length. Data are expressed as percentage of the control group (*n* = 10). #### *p* < 0.0001 vs. control; ** *p* < 0.01, *** *p* < 0.001 and **** *p* < 0.0001 vs. the MPTP.

To further validate the anti-Parkinson’s disease activity of the three peptides, FLVKLPMFM (P3), KSTELLI (P6), and SDSLSEILIS (P7), samples with gradient concentrations (12.5, 25, and 50 μg/mL) were prepared to evaluate their inhibitory effects on MPTP-induced dopaminergic neuronal loss in lines *Vmat2: GFP* zebrafish. MPTP treatment significantly reduced the length of dopaminergic neurons (*p* < 0.0001). The positive control drug, levodopa, significantly promoted the development of dopaminergic neurons in zebrafish. Similarly, the three peptides also significantly alleviated MPTP-induced damage to dopaminergic neurons in the midbrain region of zebrafish (Figure 4).

### 2.5. Effect of Peptides on Blood Vessel Development in PD-like Zebrafish

**Figure 5:**
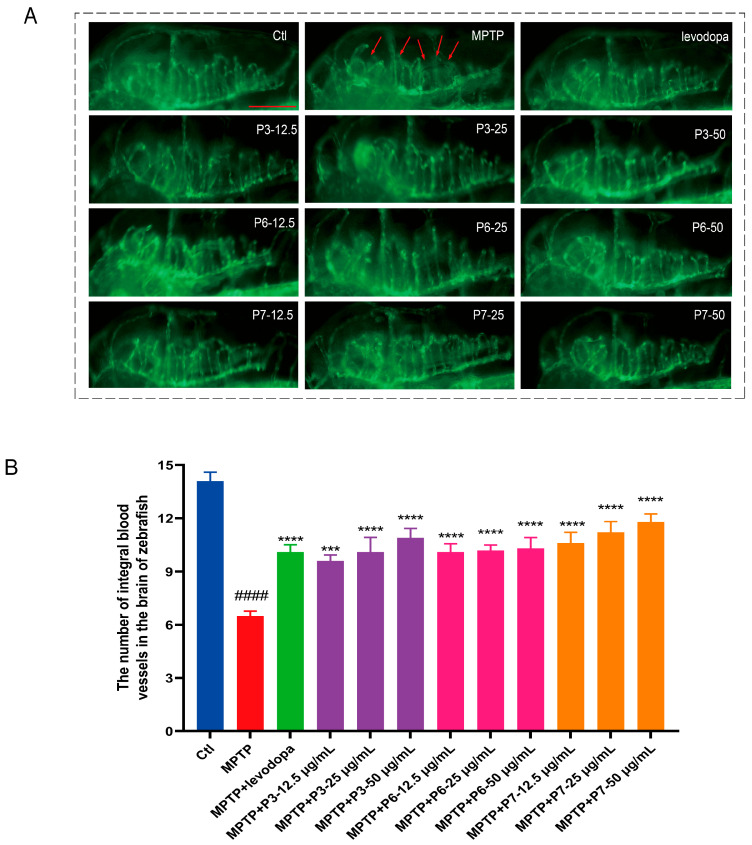
The protective effects of active peptides on blood vessels in zebrafish. (**A**) Representative fluorescence images of *Fli1:GFP* zebrafish in different treatment groups, red arrows indicate the loss of cerebral vessels. The red line is the scale bar, 200 μm; (**B**) the number of integral blood vessels in the brain (*n* = 10). #### *p* < 0.0001 vs. control; *** *p* < 0.001 and **** *p* < 0.0001 vs. the MPTP.

The protective effects of the three peptides against MPTP-induced vascular impairment were assessed using *Fli1:GFP* transgenic zebrafish. MPTP exposure resulted in a significant loss of cerebral vasculature, as marked by the red arrows in Figure 5. In contrast, co-treatment with the peptides at concentrations of 12.5, 25, and 50 μg/mL effectively attenuated this MPTP-induced vascular loss and disorganization, demonstrating a clear dose-dependent protective effect.

### 2.6. Effect of Peptides on Behavioral Analysis in PD-like Zebrafish

**Figure 6:**
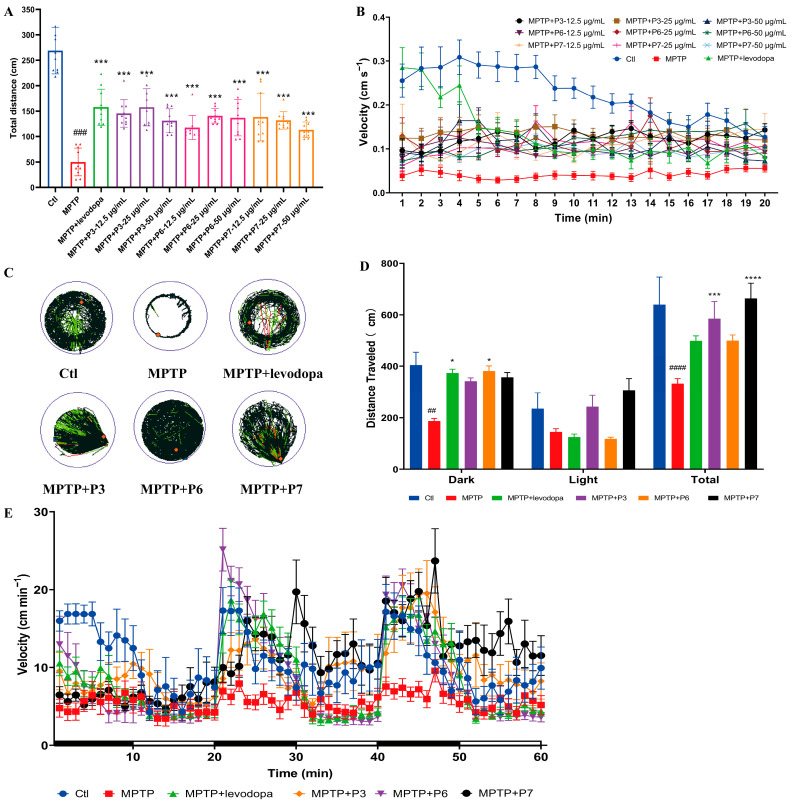
The relieving effect of three peptides on PD-like behavior in zebrafish larvae. (**A**) The total distance traveled of each zebrafish was analyzed by zeblab software (Ver 2.21) (*n* = 10). ### *p* < 0.001 vs. control; *** *p* < 0.001 vs. the MPTP. (**B**) Average swimming speed of zebrafish larvae with different treatments. Average speed in every 1 min was calculated. (**C**) Motion track, Low-speed (v < 2 cm/s) movement is represented in black lines, medium-speed (2 cm/s < v < 5 cm/s) movement is depicted in green lines, and high-speed (v > 5 cm/s) movement is represented in red lines. (**D**) Average swimming speed of zebrafish larvae responds to stimuli. Black and white band areas on the x-axis indicate dark and light periods, respectively. ## *p* < 0.01 and #### *p* < 0.0001 vs. control; * *p* < 0.05, *** *p* < 0.001 and **** *p* < 0.0001 vs. the MPTP. (**E**) The total distance, dark and light periods, and distance traveled of each zebrafish were analyzed by zeblab software (*n* = 10).

To evaluate whether co-treatment with the peptides ameliorates MPTP-induced locomotor impairment, a behavioral assessment was performed on zebrafish larvae at 120 hpf. Compared to the control group, a significant reduction in the total distance traveled was observed in the MPTP-treated group (Figure 6A). In contrast, co-treatment with the levodopa and peptides reversed this MPTP-induced decline in total distance traveled (Figure 6A). Similarly, the average swimming velocity showed a parallel trend, with MPTP exposure reducing speed and peptide co-treatment effectively restoring (Figure 6B). These results indicate that the peptides exert a protective effect on locomotor function.

In addition, a light-dark locomotion assay was employed to detect changes in the light sensitivity of zebrafish following peptide treatment using a uniform concentration of 25 μg/mL, in order to assess the potential of the peptides to improve sensory dysfunction. The movement trajectories of zebrafish larvae over 60 min differed noticeably among groups (Figure 6C). Larvae in the control group sharply increased their locomotion during dark periods and progressively reduced it, whereas MPTP-treated zebrafish did not show such a pronounced increase in movement. The trajectories of peptide-treated zebrafish were similar to those of the control group (Figure 6D).

Subsequent statistical analysis of the total distance traveled under alternating light-dark conditions revealed that, compared with the control group, MPTP exposure led to a significant decline in locomotor capacity upon environmental stimulation (*p* < 0.0001), with a particularly marked reduction in movement during dark periods. All three peptides enhanced locomotor activity in the dark. Specifically, the P6 peptide showed a light response pattern similar to that of the positive control levodopa, and the total distance traveled during dark periods was significantly higher than that in the model group (*p* < 0.05). Moreover, both P3 and P7 significantly restored overall locomotion under alternating light-dark conditions to levels comparable to the control group (*p* > 0.05) (Figure 6E).

In summary, our findings demonstrate that these three peptides significantly ameliorate the MPTP-induced decrease in locomotor activity in zebrafish, with P3 and P7 showing more pronounced recovery effects under alternating light-dark stimulation.

### 2.7. Effect of Peptides on the Contents of PD-Related Indicators in PD-like Zebrafish

**Figure 7:**
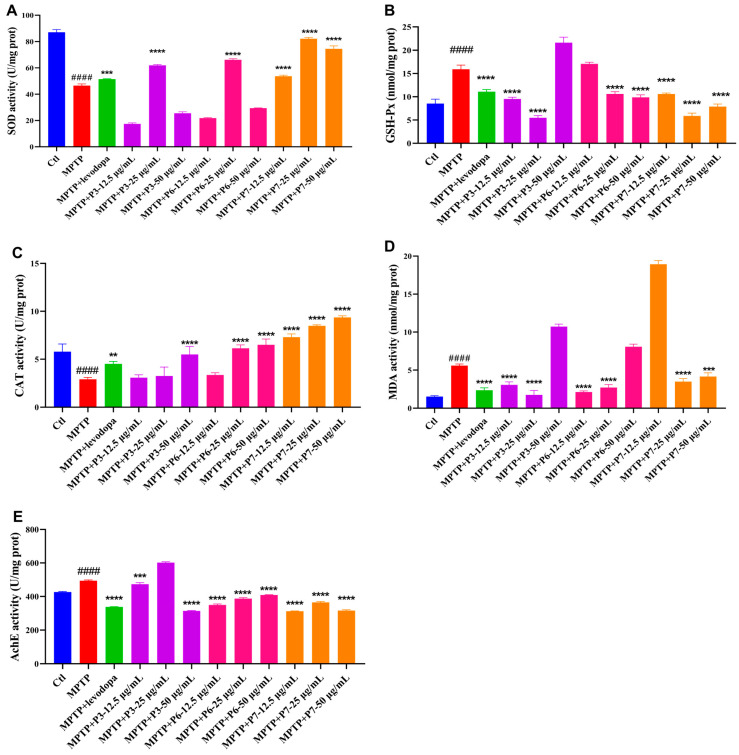
Effect of the three peptides on the PD-related indicators. (**A**) SOD activity; (**B**) GSH-Px activity; (**C**) CAT activity; (**D**) MDA content; (**E**) AchE activity. (*n* = 10). #### *p* < 0.0001 vs. control; ** *p* < 0.01, *** *p* < 0.001 and **** *p* < 0.0001 vs. the MPTP.

To further investigate the therapeutic potential of the three novel peptides against Parkinson’s disease progression, we measured the levels of key PD-related indicators—including superoxide dismutase (SOD), malondialdehyde (MDA), glutathione peroxidase (GSH-Px), catalase (CAT), and acetylcholinesterase (AChE)—in zebrafish. As shown in Figure 7, MPTP treatment significantly decreased SOD and CAT activities while increasing MDA, GSH-Px, and AChE levels compared with the blank control. Levodopa co-treatment reversed these alterations.

Among the peptides, P7 robustly elevated SOD and CAT activities. P3 and P6 significantly increased SOD activity at medium concentrations, thereby reversing the MPTP-induced decrease in enzyme activity. Furthermore, P3 significantly reversed the MPTP-induced decline in CAT activity only at high concentrations, while P6 enhanced CAT activity at both medium and high concentrations. P7 effectively normalized the compensatory rise in GSH-Px activity. P6 restored GSH-Px levels at medium and high doses, whereas P3 showed a biphasic response: it reversed the increase at low and medium concentrations but markedly overstimulated GSH-Px production at a high concentration. With regard to lipid peroxidation, P7 significantly suppressed MDA generation at all but the lowest concentration. P3 and P6 reduced MDA levels only at low and medium doses. Furthermore, P6 and P7 consistently inhibited AChE activity, while P3 produced significant inhibition solely at low and high concentrations.

### 2.8. RNA-Seq Analysis of PD-like Zebrafish Treated with Three Activity Peptides

**Figure 8:**
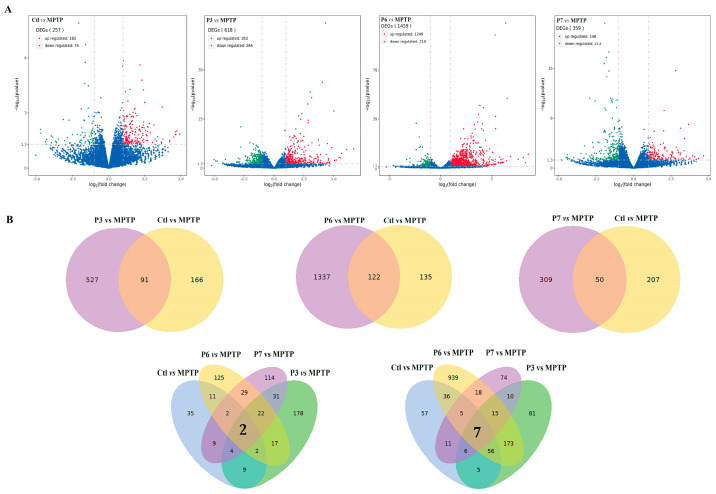

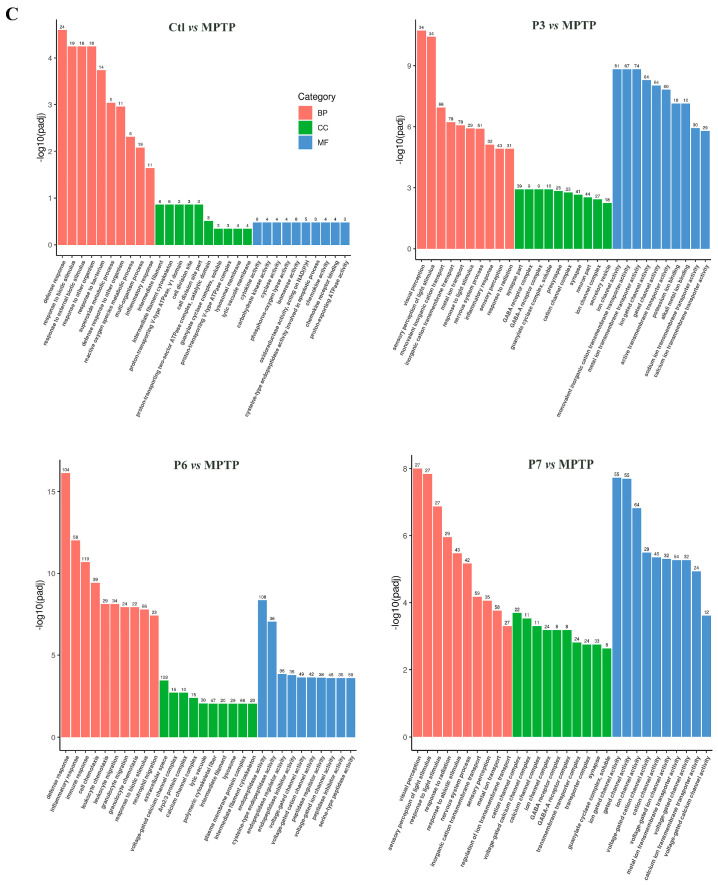

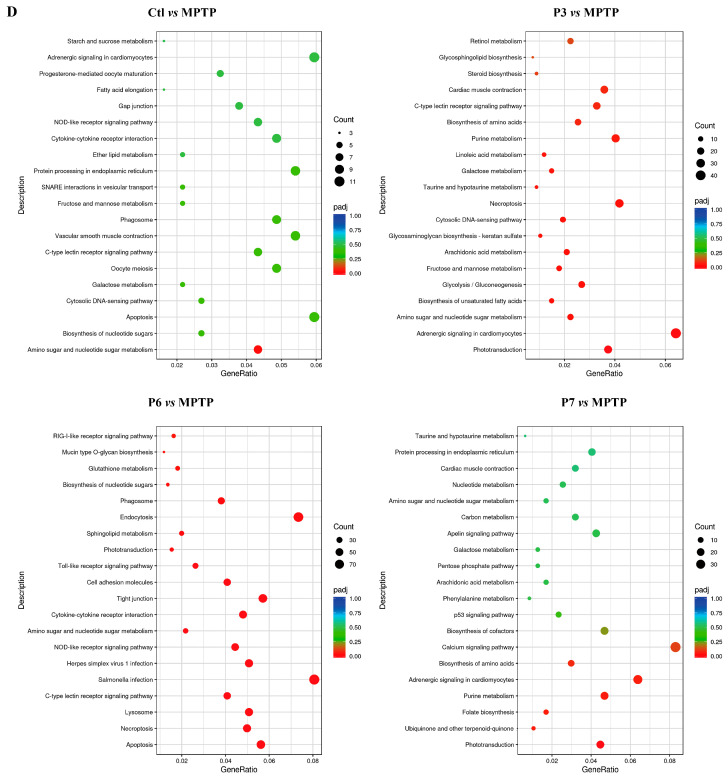
The RNA-Seq analysis of PD-like zebrafish treated with three activity peptides. (**A**) Volcanic map showing the differential gene expression distribution; (**B**) Venn diagram of common and specific DEGs between different groups; (**C**) GO functional enrichment analysis of the DEGs; (**D**) KEGG pathway enrichment of DEGs.

To elucidate the mechanism of action of three anti-PD activity peptides on zebrafish, RNAseq was performed. Transcriptomic analysis following treatment with peptides revealed distinct clustering of gene expression profiles between treated and MPTP groups, as visualized in the volcano map (Figure 8A). Genes with *p* values < 0.05 and |log2 (fold change)| > 1 were classified as differentially expressed genes (DEGs). The RNA-seq identified 257 DEGs, including 183 up-regulated genes and 74 down-regulated genes, in the MPTP group compared with the blank control group. A total of 618 DEGs, comprising 353 up-regulated genes and 265 down-regulated genes, were identified in the P3 peptide treated group compared with the model group, a total of 1459 DEGs, including 1249 up-regulated genes and 210 down-regulated genes, were identified in the P6 peptide treated group compared with the model group, and a total of 359 DEGs, including 146 up-regulated genes and 213 down-regulated genes, were identified in the P7 peptide treated group compared with the model group.

Subsequently, Venn analysis was performed to identify the overlapping DEGs between each peptide treatment group (vs. the MPTP model) and the control group (vs. the MPTP model) (Figure 8B and Figure S2). A total of 122 common DEGs were shared between the P6-treated vs. MPTP and control vs. MPTP comparisons, of which 121 showed consistent expression trends (104 up-regulated and 17 down-regulated). Similarly, 91 common DEGs were identified for the P3 comparison, all exhibiting consistent directionality (74 up-regulated and 17 down-regulated). For the P7 comparison, 50 common DEGs were found, with 29 up-regulated and 17 down-regulated genes following the same trend. From the intersections of all four comparison sets, 9 core common DEGs were identified, comprising 7 consistently up-regulated and 2 consistently down-regulated genes. These core genes are potentially associated with key biological processes such as immune-inflammatory response, apoptosis, and metabolism.

Gene Ontology (GO) enrichment analysis of the DEGs revealed their significant functional attributes across three primary categories: biological processes (BP), molecular functions (MF), and cellular components (CC). The top 30 significantly enriched GO terms are displayed in Figure 8C. Specifically, MPTP treatment significantly affected BPs related to immunity, defense responses, and reactive oxygen species metabolism. Enriched MFs were primarily associated with inflammation, oxidative stress, metabolism, and apoptosis, while CC terms included intermediate filaments, lysosomal membranes, and V-type ATPase complexes.

GO analysis further delineated distinct functional profiles for each peptide. The P6 peptide was predominantly enriched in BPs involving immune activation and inflammatory response, targeting structures such as ion channels, cytoskeletal components, and protein degradation machinery to modulate protease systems and ion channel activity. The P3 peptide showed primary enrichment in neurotransmitter receptor regulation and inflammatory response, acting on synapses, GABA-A receptors, ion channels, and secretory vesicles to modulate ion channel activity and regulate neural network homeostasis. The P7 peptide exhibited a functional profile similar to P3, with enrichment concentrated in neural signal transduction processes, where it acts on synapses and associated signaling complexes to modulate ion channel activity. The Gene set enrichment analysis (GSEA) further supported these findings. Comparative analysis revealed distinct enrichment patterns: the Ctl vs. MPTP group showed significant enrichment in processes related to proteostasis, metabolism, and the regulation of inflammatory responses. In contrast, the P6 vs. MPTP comparison was markedly enriched in reactive oxygen species metabolism and inflammatory responses while the P3 vs. MPTP comparison was significantly enriched for pathways involving apoptosis and autophagy. The P7 vs. MPTP group exhibited significant enrichment in pathways associated with cellular redox homeostasis and immune responses. (Figure S3).

The Kyoto encyclopedia of genes and genomes (KEGG) pathway analysis was conducted to identify the pathways involved. As shown in Figure 8D, comparative KEGG analysis of the DEGs between the control and MPTP-treated groups revealed significant enrichment in carbohydrate metabolism pathways, along with pathways linked to endoplasmic reticulum homeostasis, oxidative stress, and apoptosis. For the P6 peptide, a high number of DEGs were enriched in multiple pathways associated with immune and inflammatory responses, cell death, metabolism, signal transduction, and oxidative stress. In contrast, the P3 peptide showed significant pathway enrichment primarily related to metabolism, signal transduction, cell death, and oxidative stress. The P7 peptide demonstrated significant enrichment in signal transduction-related pathways, with concurrent involvement in metabolism, oxidative stress, and apoptosis. The above results indicate that modulation of metabolism, oxidative stress, inflammation, and apoptosis constitutes a common mechanism underlying the anti-Parkinson’s activity of these peptides. Furthermore, the action of the P6 peptide appears to be more focused on anti-inflammatory and immunomodulatory effects, whereas the P3 peptide shows a greater emphasis on the regulation of metabolism.

### 2.9. Effect of Three Activity Peptides on the Gene Expression Levels of Zebrafish

Combined with the results of GO and KEGG enrichment analyses, the DEGs related to the metabolism, oxidative stress, inflammation and apoptosis were selected for Quantitative reverse transcription polymerase chain reaction (qRT-PCR).

**Figure 9:**
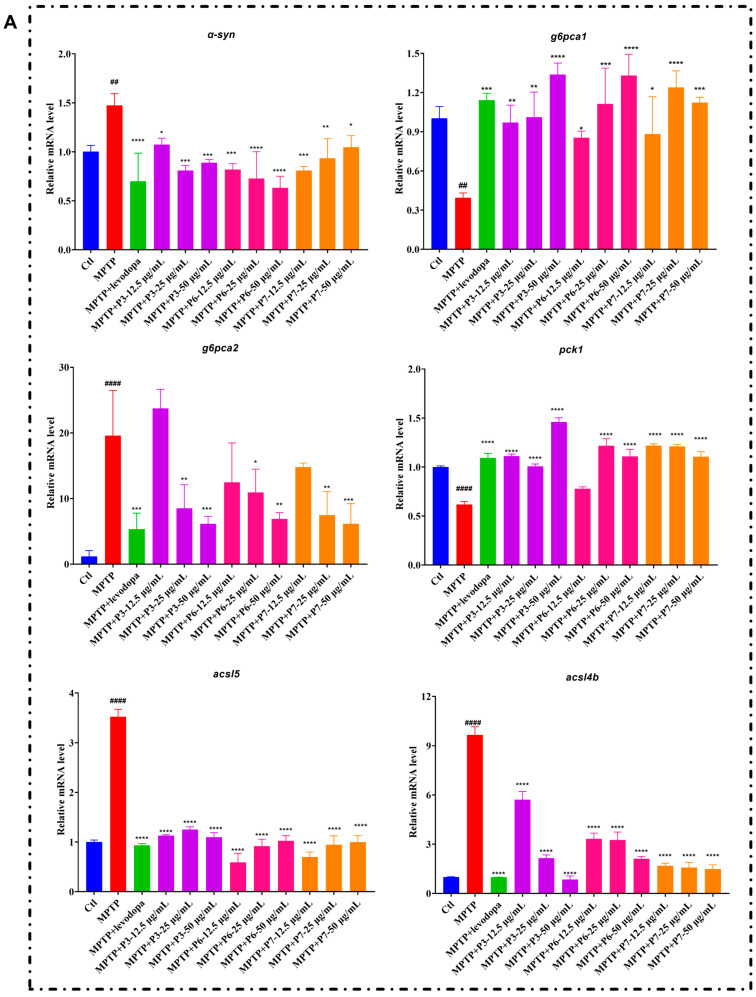

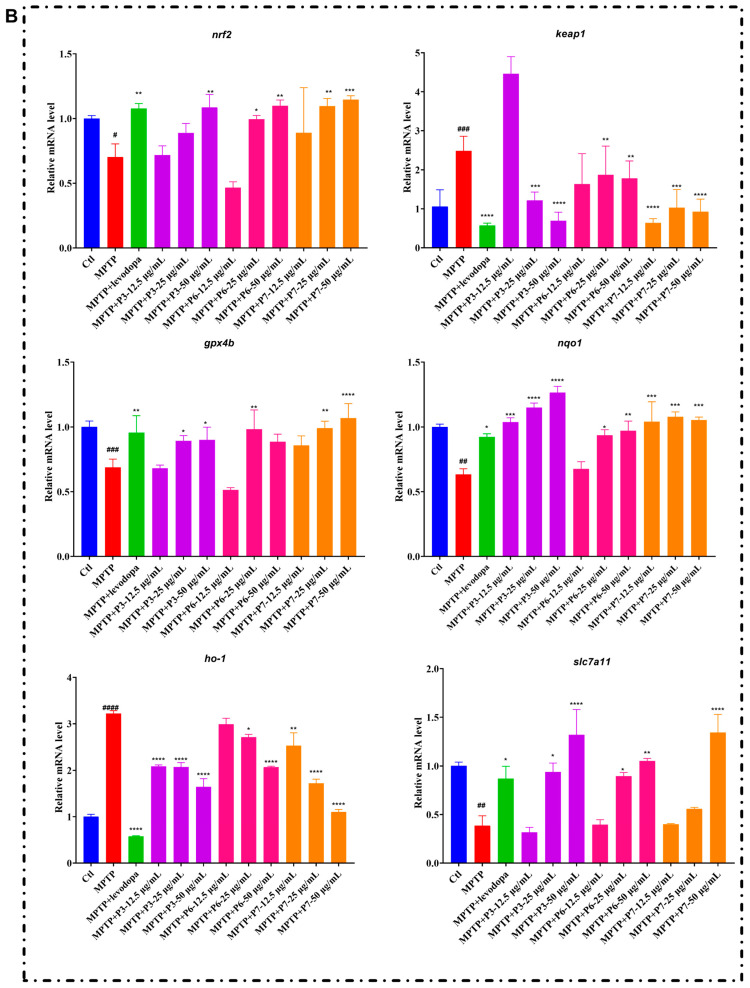

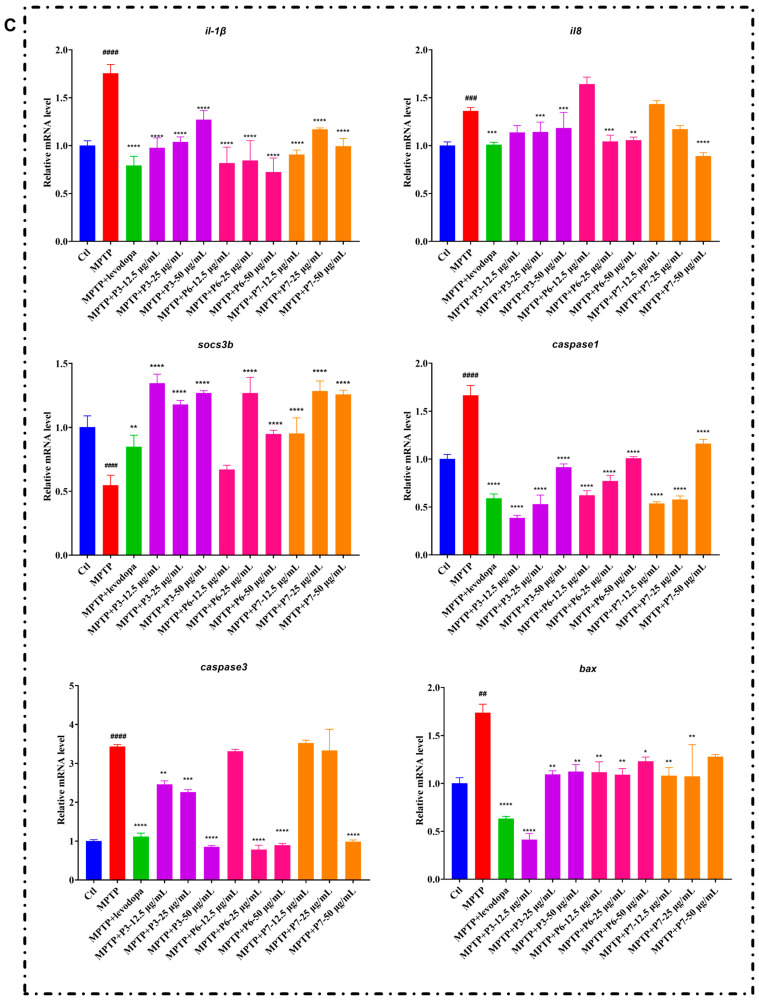
Effects of peptides on the expression of gene related to PD. (**A**) Metabolism-related genes; (**B**) Oxidative stress-related genes; (**C**) inflammation and apoptosis-related genes. (*n* = 60). # *p* < 0.05, ## *p* < 0.01, ### *p* < 0.001 and #### *p* < 0.0001 vs. control; * *p* < 0.05, ** *p* < 0.01, *** *p* < 0.001 and **** *p* < 0.0001 vs. the MPTP.

Figure 9A shows the mRNA expression levels of metabolism-related genes. Compared to the blank control group, the model group exhibited significant up-regulation of *α-syn*, *g6pca2*, *acsl5*, and *acsl4b*, and significant down-regulation of *g6pca1* and *pck1*. The positive control, levodopa, notably reversed these MPTP-induced alterations. Similarly, treatment with all three peptides significantly counteracted the abnormal expression of the above genes.

The expression levels of oxidative stress-related genes are shown in Figure 9B. The model group showed significant down-regulation of *nrf2*, *gpx4a*, *nqo1* and *slc7a11*, and significant up-regulation of *keap1* and *ho-1* compared to the control. Levodopa significantly reversed these MPTP-induced changes. Consistent with the antioxidant enzyme activity results, P7 demonstrated the most comprehensive regulatory capacity, significantly reversing the abnormal expression of all the genes tested. P6 was able to reverse the abnormal expression only at medium or high concentrations. P3 exhibited a stronger regulatory effect specifically on the downstream genes *nqo1* and *ho-1*, but could only significantly restore the expression of *nrf2*, *keap1*, *gpx4b*, and *slc7a11* at medium or high concentrations.

The effects of the peptides on inflammation- and apoptosis-related gene expression are presented in Figure 9C. The model group showed significant up-regulation of *il-1β*, *cxcl8a*, *caspase1*, *caspase3*, and *bax*, and significant down-regulation of *socs3b* compared to the blank control. Treatment with levodopa or any of the three peptides significantly reversed these MPTP-induced changes. Notably, P6 and P3 demonstrated a more comprehensive regulatory effect on these inflammation- and apoptosis-related genes.

## 3. Discussion

*Rapana venosa* is rich in protein and represents a significant potential source of bioactive peptides. However, bioactive peptides generally need to be liberated from their parent proteins to exert their functions [27]. Enzymatic hydrolysis is an effective method for peptide release, and the type and efficiency of the protease(s) used directly influence the peptide profile, yield, and bioactivity of the hydrolysate [28]. Studies have shown that a sequential hydrolysis strategy employing pepsin followed by trypsin often yields a greater quantity of peptides with enhanced bioactivity [29]. Therefore, to ensure the release of peptides with higher activity and greater stability, this study employed a simulated gastrointestinal digestion protocol to process *Rapana venosa* protein [30].

MPTP is widely used to induce Parkinson’s disease (PD) models, as it causes selective degeneration of dopaminergic (DA) neurons in the substantia nigra, recapitulating Parkinsonian symptoms [31]. Its mechanism primarily involves its conversion by monoamine oxidase B into the toxic metabolite MPP+, which inhibits mitochondrial respiratory chain complex I [32]. The loss of dopaminergic neurons in PD is often preceded by synaptic dopamine depletion and the accumulation of proteinaceous aggregates [33]. Therefore, assessing the mitigation of DA neuronal damage in zebrafish models can be used for the early and rapid screening of anti-PD active components.

To further discover potential anti-PD peptides from the *R. venosa* hydrolysate, ultrafiltration and gel filtration chromatography were employed for separation and purification. The fraction with the lowest molecular weight cut-off (Fr4, MW < 3 kDa) showed the most significant activity in mitigating DA neuronal damage. Similar trends have been reported in previous studies on the hydrolysis of proteins from sources like Ulva prolifera [34] and oyster [35]. Gel filtration chromatography is an efficient method for separating and enriching peptides based on molecular weight differences [36]. Consequently, the Fr4 fraction obtained from ultrafiltration was further separated into two sub-fractions by gel filtration. The sub-fraction with a relatively lower molecular weight demonstrated higher DA neuroprotective activity. This supports the view that peptides with low molecular weight often exhibit higher bioactivity than their high molecular weight counterparts [37].

Mass spectrometry is frequently employed for peptide structure identification due to its advantages of high efficiency, sensitivity, reliability, and reproducibility [36]. Numerous studies have shown that bioactive peptides typically consist of 2–20 amino acid residues [38]. The peptides identified from the Fr4-F2 sub-fraction all comprised ≤10 amino acid residues, suggesting their potential bioactivity. Molecular docking results also confirmed the anti-PD potential of these peptides, as they exhibited strong binding capabilities to DDC and α-syn, proteins closely associated with PD pathogenesis and therapy. Furthermore, peptides like FLVKLPMFM showed higher binding affinity to MAO-B than the positive control drug levodopa. The BIOPEP-UWM database has recently become a popular tool in bioactive peptide research, especially for peptides derived from food sources that may prevent chronic diseases [39]. Comparing sequences with reported, functionally validated peptides and predicting potential activities via this database can facilitate the rapid screening of bioactive peptides [40]. Based on screening with the BIOPEP-UWM database, we further identified three novel, previously unreported anti-PD active peptides: FLVKLPMFM (P3), KSTELLI (P6), and SDSLSEILIS (P7), which demonstrated significant DA neuroprotective activity in the PD zebrafish model.

Subsequently, we further validated the anti-PD activity of the three peptides through a multi-dimensional analytical framework integrating molecular structure, tissue microenvironment, and overall function. Molecular docking results indicated that all three peptides could stably bind to key PD target proteins (DDC, α-syn, and MAO-B). Interaction with DDC might modulate the dopamine biosynthesis pathway or inhibit its peripheral metabolism, potentially affecting the efficacy of levodopa therapy or endogenous dopamine homeostasis [41]. Binding to α-synuclein could directly interfere with its pathological oligomerization or fibrillation processes—a core pathological event in PD—thereby helping to alleviate the ensuing oxidative stress, neuronal apoptosis, and neuroinflammation [42,43]. The potential inhibition of MAO-B could directly reduce the catabolic breakdown of dopamine and the concomitant generation of reactive oxygen species, thus protecting dopaminergic neurons and suppressing apoptosis [44]. The comprehensive analysis of the interaction patterns from molecular docking provides a structural basis for the potential multi-target synergistic therapeutic effects of these peptides.

Damage to dopaminergic neurons is the primary cause of the characteristic motor symptoms of PD, including resting tremor, rigidity, bradykinesia, and postural instability [45,46]. In this study, all three peptides significantly alleviated MPTP-induced dopaminergic neuronal loss. Notably, peptide treatment also concurrently promoted the repair and normalization of the cerebral vascular network. Abnormalities in the vascular system and its regional blood supply are considered important factors in the pathogenesis of brain disorders [47]. Studies have shown that promoting cerebral angiogenesis helps inhibit neuronal apoptosis and promote neurite outgrowth, thereby mitigating the onset and progression of PD [48]. Our results further suggest that these three peptides may provide crucial microenvironmental support for neuronal survival by maintaining the structural and functional integrity of the “neurovascular unit”, leading to a significant reduction in dopaminergic neuronal damage. These improvements at the level of neuronal structure and the brain microenvironment ultimately translated directly into a significant recovery in zebrafish locomotor behavior, as measured by total movement distance and swimming velocity.

In addition to motor disorders, PD encompasses various non-motor deficits, such as sensory dysfunction and cognitive impairment. It has been reported that zebrafish larvae exhibit a significant increase in locomotor activity when illumination changes from light to dark. Impaired visual sensitivity can lead to a lack of response to such light changes [49]. This study further demonstrated that the three peptides could alleviate visual-related behavioral deficits in PD zebrafish. Among them, the P6 peptide exhibited an action mode similar to levodopa, while the P3 and P7 peptides showed more similar modes of action and function, and they demonstrated a stronger effect in improving behavioral responses to light sensitivity stimuli. This behavioral phenotypic result corresponds with the significant enrichment of the phototransduction signaling pathway in the KEGG analysis.

Studies have confirmed that elevated oxidative stress and pro-inflammatory responses occur in the early stages of PD [50]. These early events can trigger further metabolic imbalances (involving glucose, lipid, amino acid, and iron metabolism), exacerbating the overproduction of reactive oxygen species. This, in turn, drives a series of pathological processes including neuroinflammation, abnormal protein aggregation, mitochondrial dysfunction, and decreased dopamine levels, ultimately leading to insufficient cellular energy supply, dysregulation of neurotransmitter systems, aberrant aggregation and phosphorylation of α-synuclein, and loss of dopaminergic neurons [51,52]. Biochemical analysis in this study showed that treatment with the three peptides effectively reversed the MPTP-induced imbalance of oxidative stress. This was evidenced by increased activities of SOD and CAT, modulated activity of GSH-Px, reduced levels of the lipid peroxidation product MDA, and alleviation of abnormally elevated AChE activity. Concurrently, transcriptomic analysis clearly revealed significant alterations in glucose metabolism-related pathways in all peptide-treated groups compared to the MPTP model group. These results collectively demonstrate the comprehensive regulatory capacity of the peptides on the organism’s metabolic state, oxidative stress levels, and inflammatory responses.

Comprehensive analysis of the transcriptomic data and q-PCR validation results further revealed the specific mechanistic tendencies of the three peptides’ anti-PD activities. The P6 peptide was significantly enriched in inflammation-related signaling pathways, strongly suggesting that one of its core mechanisms involves modulating neuroinflammation. The action of the P3 peptide was more focused on regulating pathways related to cellular metabolism and neurotransmitter receptors, pointing to its role in improving mitochondrial function and alleviating oxidative damage. The mechanism of action of the P7 peptide showed some similarity to that of P3 but also distinct differences; it primarily affected intracellular signal transduction pathways, participated in metabolic regulation, and demonstrated strong antioxidant functionality.

The results presented above demonstrate that the three anti-PD active peptides identified from *Rapana venosa* protein hydrolysate exert protective effects across multiple pathophysiological tiers, from molecular damage and cell death to tissue function and overall animal behavior. The study reveals a unique multi-target and synergistic mechanism of action, which distinguishes these peptides from conventional single-target therapeutics.

## 4. Materials and Methods

### 4.1. Materials

Live *Rapana venosa* samples were purchased from a seafood market in Jinan, China. The samples were rinsed with deionized water, followed by the removal of the shell and operculum to collect the soft body tissues (including meat and visceral mass). The collected tissue was then stored at −80 °C until further analysis.

### 4.2. Reagents and Animals

Methyl-4-phenyl-1,2,3,6-83 tetrahydropyridine (MPTP), methylene blue and tricaine were purchased from Sigma-Aldrich (Shanghai, China). The superoxide dismutase (SOD), catalase (CAT), glutathione peroxidase (GSH-Px), malondialdehyde (MDA) and acetylcholinesterase (AchE) activity assay kits were purchased from Wuhan Servicebio Technology Co., Ltd. (Wuhan, China). The BCA protein concentration assay kit was obtained from Beyotime Biotechnology (Shanghai, China). The trypsin (250 U/mg), pepsin (250 U/mg), Sephadex G25 gel were purchased from Solarbio (Beijing, China). kitFastPure^®^ Cell/Tissue Total RNA Isolation Kit V2, HiScript^®^ III Reverse Transcriptase, and AceQ^®^ qPCR 88 SYBR Green Master Mix were obtained from Nanjing Vazyme Biotech Co., Ltd. (Nanjing, China). Wild-type AB strain zebrafish and transgenic zebrafish (*vmat2:GFP* and *fli1:GFP*) strain were maintained in our lab. Chemicals and reagents used for HPLC were of chromatographic grade and other chemicals and reagents were of analytical grade.

### 4.3. Zebrafish Maintenance and Embryo Handling

Adult zebrafish were maintained at 28 °C under a 14/10 h light/dark cycle and supplied with freshwater, aeration, and food. Embryos were obtained from natural spawning; they were collected within 30 min and cultured in an aquarium. All the experiments were performed in accordance with standard ethical guidelines. The procedures were approved by the Ethics Committee of the Biology Institute of the Shandong Academy of Science.

### 4.4. Preparation of Protein Hydrolysates from Rapana venosa Tissue

Enzymatic hydrolysis was performed following a previously described method [15] with some modifications. The *Rapana venosa* tissue was homogenized in phosphate-buffered saline (PBS, pH 7.2) at a ratio of 1:10 (*w*/*v*). The pH of the mixture was first adjusted to 2.0 using 1 mol/L HCl, followed by homogenization in an ice bath. The first enzymatic hydrolysis was then initiated by adding pepsin at an enzyme-to-substrate ratio of 1:50 (*w*/*w*) and incubating at 37 °C for 2 h. Subsequently, the pH was readjusted to 7.0 with mol/L NaOH. Following this, the second hydrolysis was carried out by adding trypsin at a ratio of 1:50 (*w*/*w*) and incubating at 37 °C for another 2 h. The reaction was terminated by heating in a boiling water bath for 10 min. The mixture was centrifuged at 10,000× *g* for 20 min at 4 °C. Finally, the supernatant was collected, lyophilized, and stored at −80 °C for further use.

### 4.5. Determination of the Degree of Hydrolysis

The DH was determined using the o-phthaldialdehyde (OPA) derivatization method according to Chen et al. [35]. Briefly, 100 μL of the enzymatic hydrolysate supernatant was mixed with 2 mL of freshly prepared OPA reagent. After reacting for 2 min at room temperature, the absorbance was measured at 340 nm using a UV-Vis spectrophotometer. A standard curve was constructed using L-serine for quantification.

### 4.6. Isolation and Purification of Peptide

#### 4.6.1. Ultrafiltration of Hydrolysates

The enzymatic hydrolysate supernatant of *Rapana venosa* protein was fractionated by ultrafiltration using a series of polyethersulfone membranes with nominal molecular weight cut-offs of 10, 5, and 3 kDa, following a modified method described by Ghelichi et al. [28]. Briefly, the supernatant was first filtered under pressure (0.25 MPa) through a 10 kDa membrane. The permeate from this step was subsequently passed through sequentially smaller membranes (5 kDa followed by 3 kDa). This sequential filtration generated four fractions: the initial retentate (>10 kDa), and the permeates corresponding to 5–10 kDa, 3–5 kDa, and <3 kDa. The fraction was concentrated via freeze-drying and stored at −80 °C pending bioactivity evaluation and subsequent purification steps.

#### 4.6.2. Purification of Active Peptides

The fraction that exhibited the highest anti-PD activity after ultrafiltration was purified according to the previously reported method with some modifications [53]. The lyophilized samples were dissolved in deionized water at a concentration of 50 mg·mL^−1^. After filtration through a 0.45 μm syringe filter, 5 mL of the solution was loaded onto a Sephadex G-25 gel filtration column (2.0 × 90 cm) pre-equilibrated with deionized water. The column was eluted with the same solvent at a flow rate of 30 mL·h^−1^. Fractions (10 mL each) were collected, and their absorbance at 220 nm was measured. According to the elution curve, the major fraction was enriched and lyophilized for further analysis.

### 4.7. Identification of Peptide Sequences by Nano-LC-LTQ-Orbitrap-MS/MS

The amino acid sequences of the bioactive peptides were identified using an EASY-nLC 1000 chromatography system (Thermo Fisher Scientific, Dreieich, Germany) coupled online to an LTQ Orbitrap Velos Pro mass spectrometer (Thermo Fisher Scientific), following a previously described method [54] with modifications. Briefly, the purified peptides were dissolved in ultrapure water containing 0.1% (*v*/*v*) trifluoroacetic acid at a final concentration of 0.1 mg/mL. A 2 μL aliquot was loaded onto a trap column (100 μm × 20 mm, 5 μm, C_18_) for enrichment and subsequently separated on an analytical column (75 μm × 150 mm, 3 μm, C_18_). Separation was achieved using a 60 min linear gradient of 0.1% (*v*/*v*) formic acid in water at a flow rate of 300 nL/min and a column temperature of 30 °C. The acquired MS/MS data were analyzed using Mascot version 2.3 (Matrix Science, Chicago, IL, USA) against the NCBInr database. Peptide identifications were considered significant with an expected value threshold of less than 0.05.

### 4.8. Peptide Synthesis

All peptides identified from the Fr4-F2 fraction (P1 to P14) were synthesized by QYAO BIO (ChinaPeptides) Co., Ltd. (Shanghai, China) using solid-phase peptide synthesis. Crude peptides were purified by preparative high-performance liquid chromatography to a purity of >95%.

### 4.9. Molecular Docking

Molecular docking was performed using AutoDock 4.2.6 to explore the binding interactions between identified peptides and therapeutic targets for PD (dopa decarboxylase enzyme, ASN protein and monoamine oxidase B enzyme). The crystal structures of the targets (human dopa decarboxylase, PDB ID: 1JS3; human alpha-synuclein protein, PDB ID: 3Q25; human monoamine oxidase B, PDB ID: 4A79) were retrieved from the RCSB Protein Data Bank (https://www.rcsb.org/, accessed on 1 January 2025). The three-dimensional structure of these peptides was constructed and energy-minimized using the MM2 force field in Chem3D Pro 14.0 (CambridgeSoft). Both receptor and ligand structures were subsequently prepared for docking using PyMOL (version 2.6) and AutoDockTools-1.5.7. Semi-flexible docking simulations were performed with 50 independent runs for each complex. The conformation with the most favorable (lowest) binding free energy was selected for further analysis.

### 4.10. Evaluation of the Anti-Parkinson’s Disease Activity of Peptides in MPTP-Induced PD Zebrafish

#### 4.10.1. Assessment of Dopaminergic Neuron Development

The MPTP-induced zebrafish PD model was established with reference to the method described by Xu et al. in our laboratory [55]. The protective effects of peptides on dopaminergic neurons were assessed using transgenic Tg (*vmat2:GFP*) zebrafish. Embryos (10–12 h post-fertilization, hpf) were maintained in E3 embryo medium (5 mM NaCl, 0.17 mM KCl, 0.33 mM CaCl_2_, 0.33 mM MgSO_4_) supplemented with 0.003% 1-phenyl-2-thiourea (PTU) to inhibit pigmentation and enhance optical clarity. Zebrafish larvae at 24 h post-fertilization (hpf) were randomly distributed into 24-well cell culture plates, with 10 larvae per well in 2 mL of bathing medium. They were assigned to the following groups: control, MPTP model, levodopa positive control, and peptide treatment groups. To induce the PD-like model, embryos were exposed to 50 μM MPTP. For the initial screening of anti-PD fractions and peptides, a uniform concentration of 100 μg/mL was applied. In subsequent validation experiments, the selected active peptides were tested at three different concentrations (low, medium, and high). From 24 to 96 hpf, larvae were co-treated daily with MPTP and the respective peptide (or levodopa in the positive control group at 6 μg/mL). The treatment solution was refreshed daily with new E3 medium containing the corresponding drugs to maintain constant exposure. At the experimental endpoint, ten larvae per group were randomly selected for imaging. The length of the GFP-positive dopaminergic neuron region was quantified from micrographs acquired using a Zeiss microscope (Jena, Germany).

#### 4.10.2. Assessment of Blood Vessel Development

The protective effects of peptides on the neural vasculature were evaluated using transgenic Tg (*fli1:GFP*) zebrafish. Embryo rearing, PTU treatment, grouping, and the peptide treatment regimen (including concentration settings and co-treatment with MPTP from 24 to 96 hpf) were performed as described in Section 4.10.1. The treatment medium was also refreshed daily. Ten larvae per group were randomly selected for observation and imaging. The loss of the cerebro vasculature in the zebrafish brain was observed, photographed, and analyzed using a Zeiss microscope (Jena, Germany).

#### 4.10.3. Behavioral Analysis

Locomotor activity was assessed to evaluate the effects of treatment with active peptides on the behavior of AB wild-type zebrafish larvae. Larvae at 120 hpf from each group were individually transferred into the wells of a 48-well plate, each containing 600 µL of embryo medium. The plate was then placed in an automated video-tracking system, and the larvae were allowed to acclimate for 15 min prior to testing. Locomotor activity was recorded for 20 min. The total swimming distance, average velocity, and movement trajectories were analyzed using ZebLab software (Viewpoint, Lyon, France).

#### 4.10.4. The Light and Dark Challenges Assay

A light-dark locomotion assay was performed to quantify the locomotor response of zebrafish larvae to sudden changes in light intensity, following an established protocol [56]. At 120 hpf, larvae from each group were individually transferred into wells of a 48-well plate containing 1000 μL of bathing medium. The light-dark cycle was programmed using ZebLab software (Viewpoint Life Sciences, Lyon, France) and consisted of a 10 min acclimation period followed by three 10 min dark and 10 min light cycles, for a total duration of 60 min. The total distance moved and swimming velocity were automatically tracked and analyzed using the ZebLab software.

#### 4.10.5. Determination of Parkinson’s Disease-Related Indicator

For biochemical analysis, fifty zebrafish larvae from each group were homogenized on ice and centrifuged (8000–10,000× *g*, 10 min, 4 °C), and the resulting supernatant was subsequently analyzed for total protein content and the activities of SOD, CAT, GSH-Px, MDA and AChE using corresponding commercial assay kits, following the respective protocols.

### 4.11. Identification of Differentially Expressed Genes in Zebrafish Using Transcriptome Sequencing

AB wild-type zebrafish larvae were grouped and treated as described in Section 4.10.1 and rinsed twice with PBS in 3 replicates per group. Three independent experiments were performed for each group to obtain biological replicates. Transcriptome sequencing analysis was carried out with the assistance of Novogene Biotechnology Co., Ltd. (Beijing, China). Genes with *p* values < 0.05 and |log2 (fold change)| > 1 were classified as DEGs. GO enrichment analysis of the DEGs was performed using the GOseq R package (Ver 1.20.0) based on the Wallenius non-central hypergeometric distribution, which adjusts for bias in gene length among DEGs. And the enrichment analyses of the KEGG pathway to identify the functions and potential expression trends of the DEGs.

### 4.12. Quantitative Real-Time RT-PCR Assay

To validate transcriptomic results, gene expression was analyzed by qRT-PCR using RNA from the same samples employed for sequencing. Total RNA was isolated using the Tissue Total RNA Isolation Kit according to the manufacturer’s instructions. The cDNA of each group was synthesized using the HiScript III RT SuperMix (Vazyme Biotech, Nanjing, China). Quantitative real-time PCR was then performed on a LightCycler^®^ 96 System (Roche, Rotkreuz, Switzerland) using ChamQ Universal SYBR qPCR Master Mix (Vazyme Biotech, Nanjing, China). β-actin was selected as an internal reference gene to normalize mRNA expression levels. The relative mRNA expression levels were calculated using the 2^−ΔΔCT^ method. The primer sequences are listed in Supplemental Table S2.

### 4.13. Statistical Analysis

All data are presented as the mean ± standard error of the mean (SEM). Statistical analyses were performed using GraphPad Prism software (version 8.3.0). One-way analysis of variance was used to analyze differences among groups, and *p* < 0.05 was considered statistically significant.

## 5. Conclusions

This study, for the first time, successfully identified three novel peptides with significant anti-PD activity, P6, P3, and P7, from *Rapana venosa* protein hydrolysates through systematic enzymatic digestion, ultrafiltration, gel filtration chromatography, and bioactivity-guided screening. Using an MPTP-induced zebrafish PD model, we demonstrated that these peptides exert comprehensive neuroprotective effects via multi-target and multi-pathway synergistic mechanisms. They effectively protected midbrain dopaminergic neurons, improved the cerebral vascular network, rescued locomotor deficits, and modulated cholinergic function. At the molecular level, the peptides coordinately upregulated cellular antioxidant defenses, restored metabolic homeostasis, and suppressed neuroinflammation and apoptosis. This work provides promising candidate molecules and a solid scientific foundation for developing novel, marine resource-based, multi-target therapeutic strategies against Parkinson’s disease.

## Figures and Tables

**Table 1:** The binding energy of identified peptides with key targets.

Ligand	Binding Energy (KJ/mol)		
	1JS3 (DDC)	3Q25 (α-syn)	4A79 (MAO-B)
levodopa	−7.2	−7.3	−6.9
ARLGLAIL (P1)	−8.1	−7.7	−6.6
FGINLIQ (P2)	−8.9	−8.5	−7.2
FLVKLPMFM (P3)	−8.3	−8.5	−7.3
GLEINLIGF (P4)	−7.7	−8.2	−7.0
GYSFTTTAER (P5)	−8.0	−8.1	−6.8
KSTELLI (P6)	−7.7	−7.3	−6.4
SDSLSEILIS (P7)	−7.4	−7.4	−6.8
LLGEILI (P8)	−8.0	−7.7	−6.6
LLIRAGL (P9)	−8.2	−8.9	−7.2
MILLGLVLMG (P10)	−7.5	−7.0	−5.6
MVLLGLVLMG (P11)	−7.3	−7.4	−5.9
MWISKQEYD (P12)	−8.4	−7.9	−6.4
NLLGLVL (P13)	−7.9	−7.8	−7.2
TNLLMILLI (P14)	−7.1	−7.4	−5.4

## Data Availability

The data presented in the current study are available on request from the corresponding author.

## Supplementary Materials

The following supporting information can be downloaded at: https://www.mdpi.com/article/10.3390/md24050180/s1, Figure S1: The molecular docking results for the ligand-receptor complex; Figure S2: Venn diagram analysis of common and specific DEGs with consistent regulatory trends between different groups; Figure S3: GSEA was conducted using GO pathways’ biological process branch as the gene sets of interest; Table S1: Screening results of potential anti-PD bioactive amino acid fragments; Table S2: The gene primers for quantitative real-time PCR.

## Abbreviations

The following abbreviations are used in this manuscript:

PD	Parkinson disease
MPTP	1-methyl-4-phenyl-1,2,3,6-tetrahydropyridine
DH	Degree of Hydrolysis
P6	KSTELLI
P3	FLVKLPMFM
P7	SDSLSEILIS
DDC	Dopa decarboxylase
α-syn	Alpha-synuclein
MAO-B	Monoamine oxidase b
SOD	Superoxide dismutase
MDA	Malondialdehyde
GSH-Px	Glutathione peroxidase
CAT	Catalase
AchE	Acetylcholinesterase
DEGs	Differentially expressed genes
GO	Gene ontology
BP	Biological processes
MF	Molecular functions
CC	Cellular components
GSEA	Gene set enrichment analysis
KEGG	Kyoto encyclopedia of genes and genomes
qRT-PCR	Quantitative reverse transcription polymerase chain reaction
