# Supplementary material for: Identification, Screening and Mechanism Analysis of Anti-Parkinson’s Disease Peptides from Rapana venosa Protein Hydrolysates
Source: Mar Drugs. 2026 May 15;24(5):180. doi: 10.3390/md24050180 (PMC13208443; doi:10.3390/md24050180)
Supplement: Supplementary file 1 [file marinedrugs-24-00180-s001.zip › marinedrugs-4301120-supplementary.pdf]

Supplementary Material

Identification, Screening and Mechanism Analysis of Anti-Parkinson's Disease Peptides from *Ramana venosa* Protein Hydrolysates

Qingzhong Wang[1], Shuqin Shao[1], Yizhuo Wang[1], Wenshuai Fan[1], Zilong Wang[1], Xuchang Liu[1], Kechun Liu[1] and Shanshan Zhang[1,*]

[1] Biology Institute, Qilu University of Technology (Shandong Academy of Sciences), Jinan 250103, China.

* Correspondence:
zhangss369@126.com (S.-S.Z.)

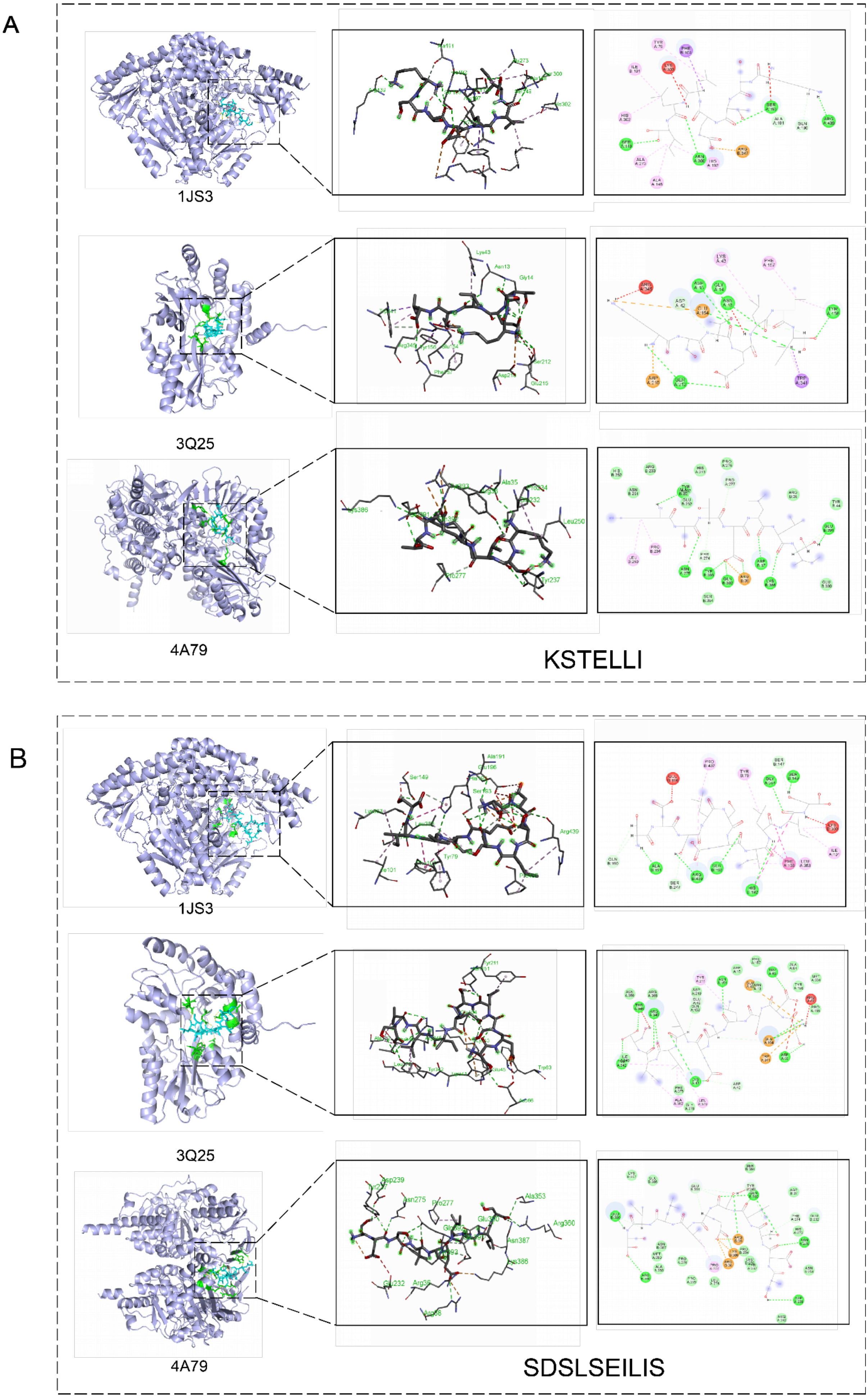
A
1JS3
3Q25
4A79
KSTELLI
B
1JS3
3Q25
4A79
SDSLSEILIS

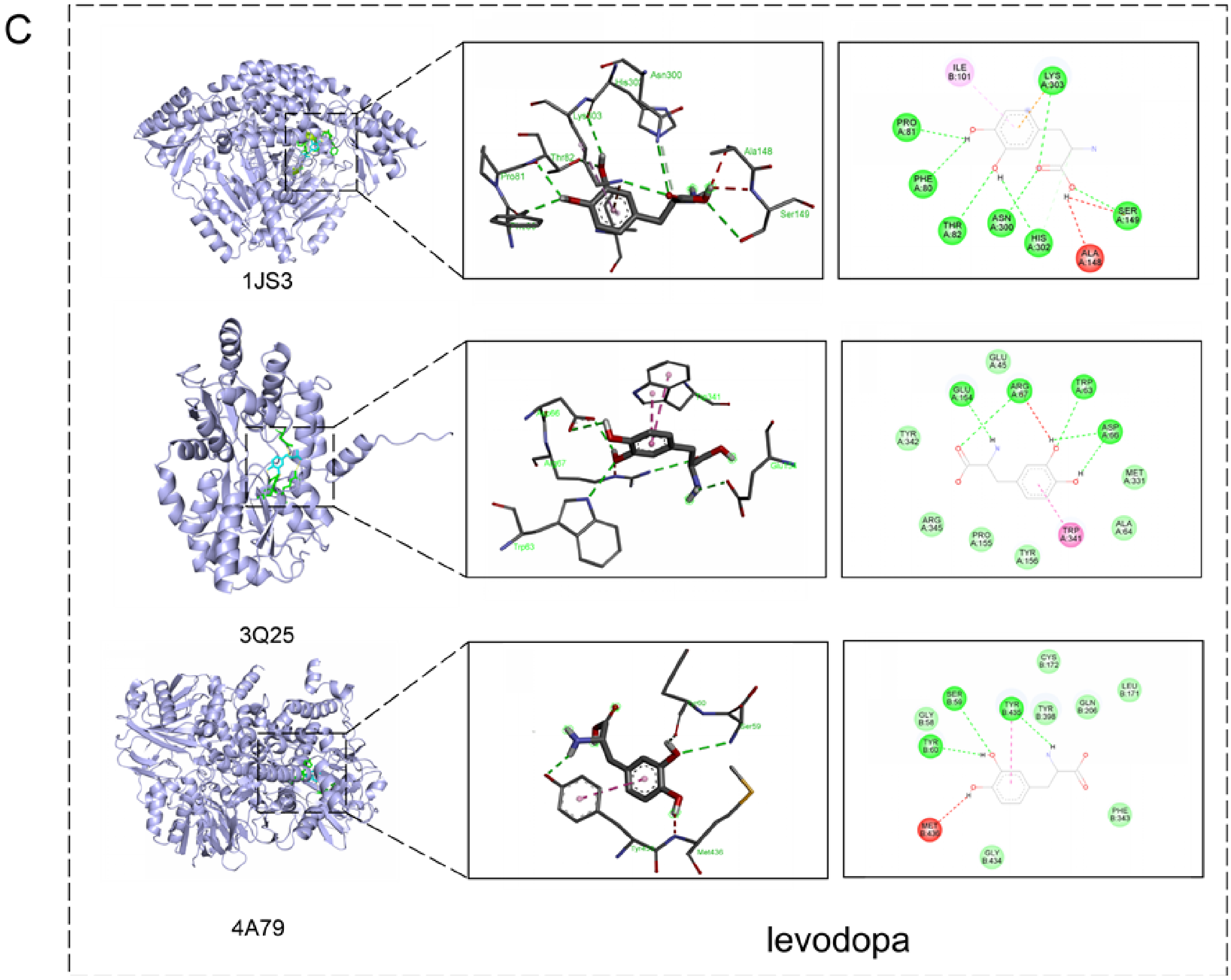

Figure. S1. The molecular docking results for the ligand-receptor complex. **(A)** KSTELLI (P6); **(B)** SDSLSEILIS (P7); **(C)** the positive control, levodopa.

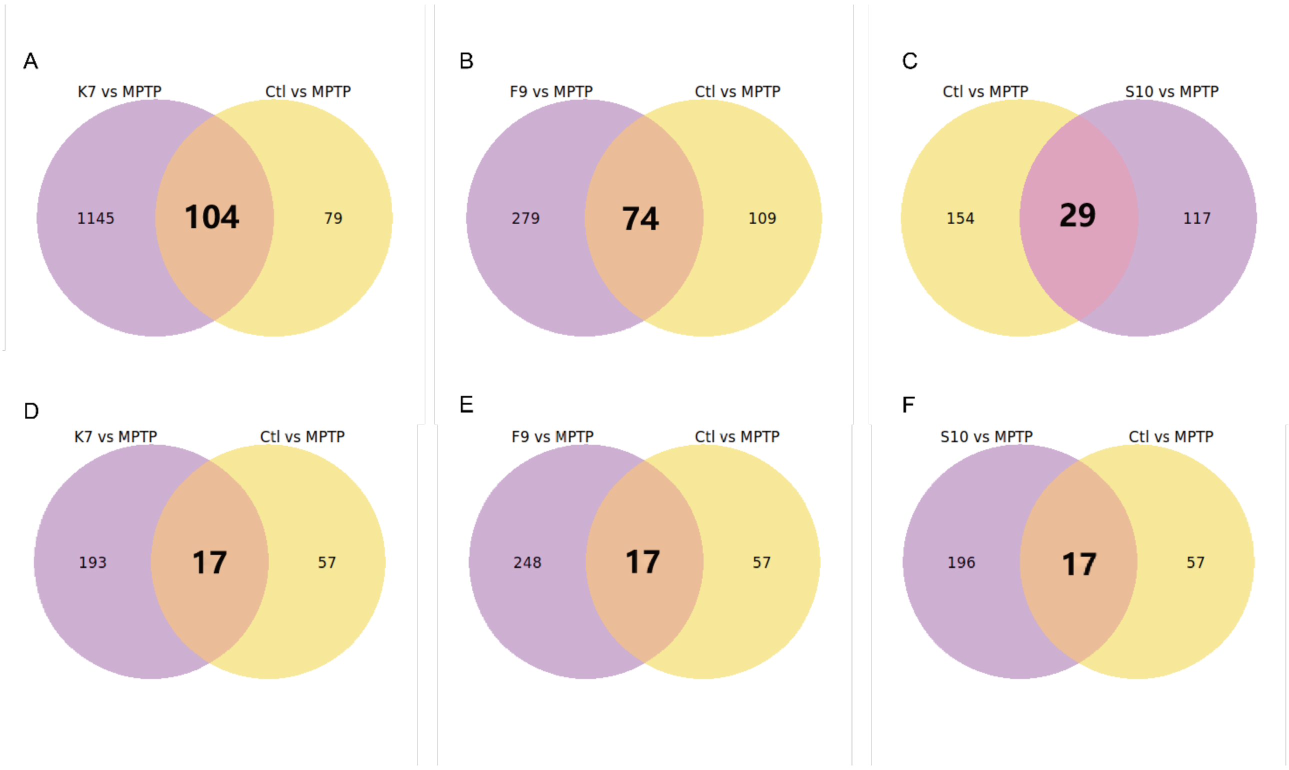

Figure. S2. Venn diagram analysis of common and specific DEGs with consistent regulatory trends between different groups. **(A)**Up-regulated genes shared between the Ctl vs. MPTP and the K7 vs. MPTP comparisons; **(B)**Up-regulated genes shared between the Ctl vs. MPTP and the F9 vs. MPTP comparisons; **(C)**Up-regulated genes shared between the Ctl vs. MPTP and the S10 vs. MPTP comparisons; **(D)**Down-regulated genes shared between the Ctl vs. MPTP and the K7 vs. MPTP comparisons; **(E)**Down-regulated genes shared between the Ctl vs. MPTP and the F9 vs. MPTP comparisons; **(F)**Down-regulated genes shared between the Ctl vs. MPTP and the S10 vs. MPTP comparisons.

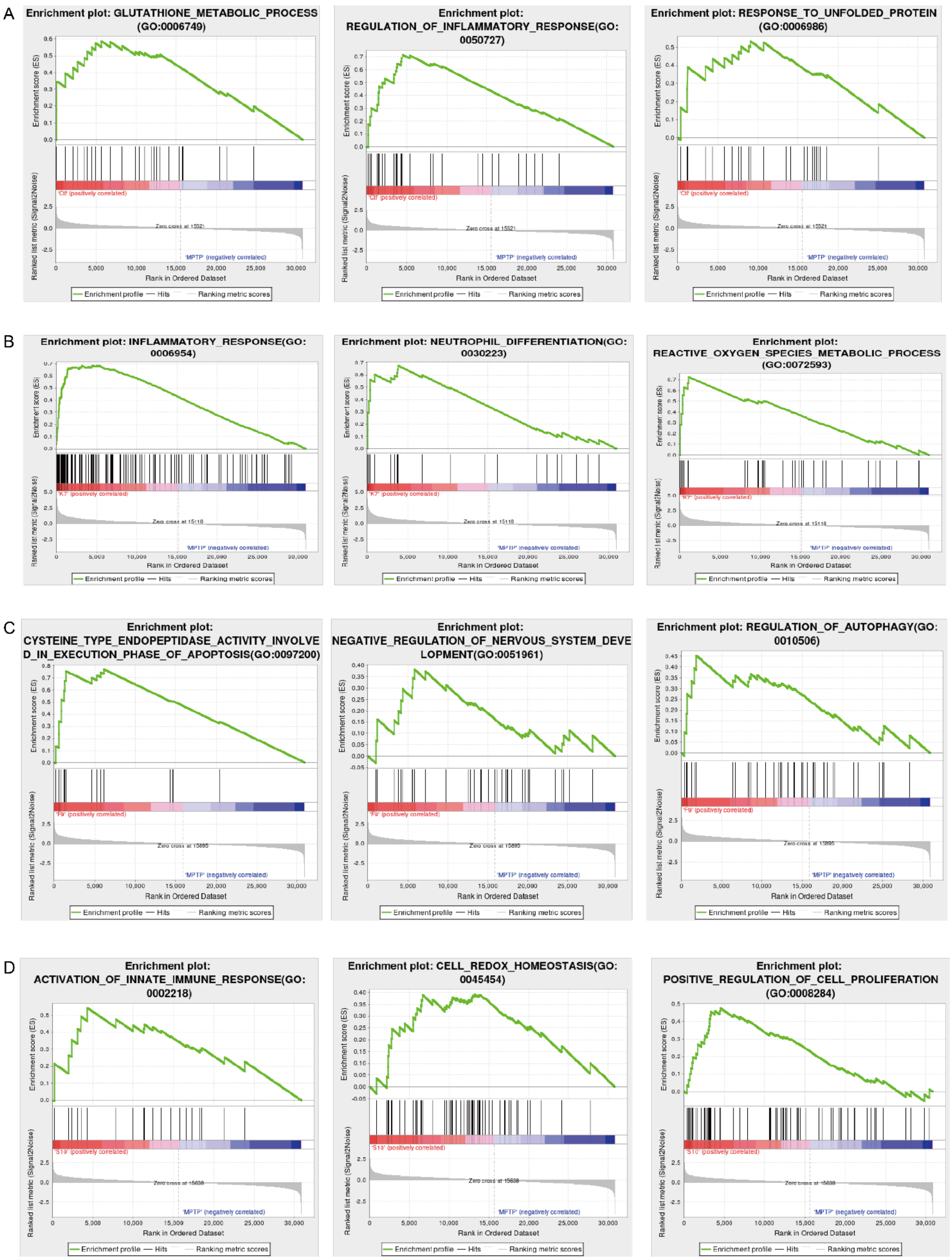

Figure S3. GSEA was conducted using GO pathways' biological process branch as the gene sets of interest. **(A)** Ctl vs. MPTP; (**B**) K7 vs. MPTP; (**C**) F9 vs. MPTP; (**D**) S10 vs. MPTP.

Table S1. Screening results of potential anti-PD bioactive amino acid fragments.

Sequence	Activity	Peptides
RL	ACE inhibitor	ARLGLAIL
IR	ACE inhibitor	LLIRAGL
MF	ACE inhibitor	FLVKLPMFM
IRA	ACE inhibitor	LLIRAGL
VK	ACE inhibitor	FLVKLPMFM
RA	ACE inhibitor	LLIRAGL
GF	ACE inhibitor	GLEINLIGF, FLVKLPMFM
KL	ACE inhibitor	FLVKLPMFM
AR	ACE inhibitor	ARLGLAIL
EY	ACE inhibitor	MWISKQEYD
EI	ACE inhibitor	SDSLSEILIS
TE	ACE inhibitor	KSTELLI
IL	ACE inhibitor	SDSLSEILIS, LLGEILI, TNLLMILLI
MW	ACE inhibitor	MWISKQEYD
ST	ACE inhibitor	KSTELLI
KLP	ACE inhibitor	FLVKLPMFM
ER	ACE inhibitor	GYSFTTTAER, SDSLSEILIS
LP	ACE inhibitor	FLVKLPMFM
YS	ACE inhibitor	GYSFTTTAER
FL	ACE inhibitor	FLVKLPMFM, LLIRAGL
VL	stimulating	FLVKLPMFM, MILLGLVLMG, MVLLGLVLMG
LV	stimulating	FLVKLPMFM
IL	stimulating	FGINLIQ, KSTELLI, LLGEILI, TNLLMILLI, MILLGLVLMG
LI	stimulating	KSTELLI, LLGEILI, LLIRAGL, NLLGLVL, MILLGLVLMG, MVLLGLVLMG, TNLLMILLI
LL	stimulating	KSTELLI
SE	stimulating	SDSLSEILIS
IL	neuropeptide	SDSLSEILIS
FL	neuropeptide	FLVKLPMFM
SL	regulating	SDSLSEILIS
EL	antioxidative	KSTELLI
VKL	antioxidative	FLVKLPMFM
IR	antioxidative	LLIRAGL
ELLI	antioxidative	KSTELLI
LPM	antioxidative	FLVKLPMFM
RA	activating ubiquitin-mediated proteolysis	LLIRAGL
LA	activating ubiquitin-mediated proteolysis	ARLGLAIL
LP	dipeptidyl peptidase IV inhibitor	FLVKLPMFM
LL	dipeptidyl peptidase IV inhibitor	KSTELLI, MILLGLVLMG, TNLLMILLI
TA	dipeptidyl peptidase IV inhibitor	GYSFTTTAER
FL	dipeptidyl peptidase IV inhibitor	FLVKLPMFM
SL	dipeptidyl peptidase IV inhibitor	SDSLSEILIS
GL	dipeptidyl peptidase IV inhibitor	GLEINLIGF

WI	dipeptidyl peptidase IV inhibitor	MWISKQEYD
AE	dipeptidyl peptidase IV inhibitor	GYSFTTTAER
EI	dipeptidyl peptidase IV inhibitor	SDSLSEILIS, GLEINLIGF
EY	dipeptidyl peptidase IV inhibitor	MWISKQEYD
GE	dipeptidyl peptidase IV inhibitor	LLGEILI
IL	dipeptidyl peptidase IV inhibitor	SDSLSEILIS, ARLGLAIL
IN	dipeptidyl peptidase IV inhibitor	GLEINLIGF
IQ	dipeptidyl peptidase IV inhibitor	FGINLIQ
IR	dipeptidyl peptidase IV inhibitor	LLIRAGL
KS	dipeptidyl peptidase IV inhibitor	KSTELLI
LI	dipeptidyl peptidase IV inhibitor	KSTELLI, SDSLSEILIS, FGINLIQ
LM	dipeptidyl peptidase IV inhibitor	TNLLMILLI
LV	dipeptidyl peptidase IV inhibitor	FLVKLPMFM, MILLGLVLMG, MVLLGLVLMG
MF	dipeptidyl peptidase IV inhibitor	FLVKLPMFM
MG	dipeptidyl peptidase IV inhibitor	MILLGLVLMG, MVLLGLVLMG
MI	dipeptidyl peptidase IV inhibitor	MILLGLVLMG
MV	dipeptidyl peptidase IV inhibitor	MVLLGLVLMG
NL	dipeptidyl peptidase IV inhibitor	GLEINLIGF
PM	dipeptidyl peptidase IV inhibitor	FLVKLPMFM
QE	dipeptidyl peptidase IV inhibitor	MWISKQEYD
TE	dipeptidyl peptidase IV inhibitor	KSTELLI
VK	dipeptidyl peptidase IV inhibitor	FLVKLPMFM
LA	dipeptidyl peptidase III inhibitor	ARLGLAIL
FL	dipeptidyl peptidase III inhibitor	FLVKLPMFM
FM	dipeptidyl peptidase III inhibitor	FLVKLPMFM

Table S2. The gene primers for quantitative real-time PCR.

Primer		Sequence
β-actin	Forward	ACCACGGCCGAAAGAGAAAT
	Reverse	GATACCGCAAGATTCCATACCC
α-syn	Forward	ATGGATGTTTTTATGAAGGGGC
	Reverse	ACGCTGTCTTTGGTCTTGCT
keap1	Forward	ATACCAACCAGACACCAACAC
	Reverse	GGTTTGTCCATCATAGCCTCC
nrf2	Forward	ATGTCTAAAATGCAGCCAAGCC
	Reverse	CGGTAGCTGAAGTCGAACAC
gpx4b	Forward	GAGATTAAGGAGTTTGCTAAAGGCT
	Reverse	CTTGGTGAAATTCCACTTGATGTTG
nqo-1	Forward	CTGGGTGGTGTGTTTGAAGAA
	Reverse	GCTGTGGTAATGCCGTAGG
ho-1	Forward	AAGCAAAGCGGCAGAGAAC
	Reverse	TGGAGCAGTCAGATGAAGTGT
slc7a11	Forward	GTATCAACTTTTCAAAGGGGAGACC
	Reverse	CACGAAATTCAAGTAAAACCAGCC
g6pca1	Forward	GCCCAGAAATGGTGCATAAATCC
	Reverse	TGAGACAAATGAAGGCCGAGAC

g6pca2	Forward	AGATCTGTGTTTGTCTCTCTCGGGT
	Reverse	ACAAACCCACAGCGAACGA
pck1	Forward	GAAGCCGCTGGTCAACAACT
	Reverse	CAGCAGTGAGTTTCCTCCGTATC
acsl4b	Forward	CTCTCACGGACCAGTCTAGTAAAAT
	Reverse	CTCCCAGAAGCACATTCACCT
acsl5	Forward	GAGAAGAAATGTGCGGAAGTCAA
	Reverse	AAGTGAGCACAGATGGAGAAATAGG
socs3b	Forward	GGTAACGCATAGTAGGCTTGACAG
	Reverse	CTGATACTGCTCCCGAGAACTGA
caspase1	Forward	ACGGCTCCTTCCCAGTGATA
	Reverse	TCAGCAAAGGAAATGGAT
caspase3	Forward	TCAGTCACGGCGATGAGGG
	Reverse	CCTCGACAAGCCTGAATAAAGAAC
il8	Forward	TCCTGGCATTTCTGACCATCAT
	Reverse	ATGCGTCGGCTTTCTGTTTC
il-1β	Forward	GTACTCAAGGAGATCAGCGG
	Reverse	CTCGGTGTCTTTCCTGTCCA
bax	Forward	GGCTATTTCAACCAGGGTTCC
	Reverse	TGCGAATCACCAATGCTGT
